# Supplementary material for: Coordinated RNA- and protein-templated synthesis of double-stranded DNA by a dual reverse transcriptase immune system
Source: bioRxiv. 2026 Jul 10:2026.05.04.722794. Originally published 2026 May 5. Preprint. [Version 2] doi: 10.64898/2026.05.04.722794 (PMC13174585; doi:10.64898/2026.05.04.722794)
Supplement: Supplement 8 [file media-8.pdf]

KEY RESOURCES TABLE

REAGENT or RESOURCE	SOURCE	IDENTIFIER
**Antibodies**		
Mouse monoclonal anti-FLAG	Sigma-Aldrich	Cat#F3165; RRID: AB_259529
**Bacterial and virus strains**		
E. coli K-12 MG1655 (sSL0810)	Yale *E. coli* Genetic Stock Center	U00096.3
E. coli NEB Turbo (sSL0410)	New England Biolabs	Cat#C2984H
E. coli BW25113 (sSL0800)	Gift of S. Tavazoie from Horizon Discovery Keio Collection[40] (Baba et al., 2006)	CGSC#7636
E. coli BW25113 Δ*sbcB* (sSL5293)	Gift of S. Tavazoie from Horizon Discovery Keio Collection[40] (Baba et al., 2006)	JW1993
E. coli BW25113 Δ*recA* (sSL6348)	Horizon Discovery Keio Collection[40] (Baba et al., 2006)	JW2669
E. coli BW25113 Δ*recB* (sSL0806)	Gift of S. Tavazoie from Horizon Discovery Keio Collection[40] (Baba et al., 2006)	JW2788
E. coli BW25113 Δ*recC* (sSL0807)	Gift of S. Tavazoie from Horizon Discovery Keio Collection[40] (Baba et al., 2006)	JW2790
E. coli BW25113 Δ*recD* (sSL0805)	Gift of S. Tavazoie from Horizon Discovery Keio Collection[40] (Baba et al., 2006)	JW2787
E. coli BW25113 Δ*sulA* (sSL6352)	Horizon Discovery Keio Collection[40] (Baba et al., 2006)	JW0941
E. coli BW25113 Δ*greA* (sSL6000)	Horizon Discovery Keio Collection[40] (Baba et al., 2006)	JW3148
Bacteriophage λ-vir	Gift from M. Laub	N/A
Bacteriophage T5	Gift from M. Laub	N/A
E. coli Rosetta2 (DE3) (sHN0002)	Novagen	Cat#71397
**Chemicals, peptides, and recombinant proteins**		
DRT3a protein	This paper	N/A
DRT3a mutant protein	This paper	N/A
DRT3b protein	This paper	N/A
DRT3b mutant proteins	This paper	N/A
SUMO protease	This paper	N/A
T7 RNA polymerase	This paper	N/A
Proteinase K for *in vitro* gel experiments	Sigma-Aldrich	Cat#3115836001
RNase A for *in vitro* gel experiments	NEB	Cat#T3018L
Proteinase K for *in vivo* and *in vitro* sequencing experiments	Thermo Fisher Scientific	Cat#EO0491
RNase A for *in vivo* and *in vitro* sequencing experiments	Thermo Fisher Scientific	Cat#EN0531
TnY transposase	This paper	N/A
TURBO DNase	Thermo Fisher Scientific	Cat#AM2238
RppH	New England Biolabs	Cat#M0356
T4 polynucleotide kinase	New England Biolabs	Cat#M0201
DNase I	New England Biolabs	Cat#M0303
Exonuclease I	New England Biolabs	Cat#M0568
MmeI	New England Biolabs	Cat#R0637
Sequencing-grade modified trypsin	Promega	Cat#V5113
SUPERase•In RNase Inhibitor	Thermo Fisher Scientific	Cat#AM2696
**Critical commercial assays**		
xGen ssDNA & Low-Input DNA Library Prep Kit	Integrated DNA Technologies	Cat#10009817
HyperLight FluorGreen dsDNA Assay Kit	APExBIO	Cat#K1603
**Deposited data**		
Hexameric DRT3b complex map	This paper	EMD-80550
Hexameric DRT3b complex coordinates	This paper	PDB: 26CZ
Raw cryo-EM images of the hexameric DRT3b complex	This paper	EMPIAR-13689
λ escaper sequencing	This paper	BioProject: PRJNA1461384
cDIP–sequencing	This paper	GEO: GSE330639
Miniprep–sequencing	This paper	GEO: GSE330641
Tn–sequencing	This paper	GEO: GSE329894
RNA–sequencing	This paper	GEO: GSE329895
RIP–sequencing	This paper	GEO: GSE329897
Immunoprecipitation-mass spectrometry	This paper	MassIVE: MSV000101674
**Oligonucleotides**		
DNA oligos (for *in vitro* transcription sequencing)	This paper	Table S4
DNA oligos (for TnY escaper sequencing)	This paper	Table S4
DNA oligos (for *in vitro* transcription)	This paper	Table S4
DNA oligos (for *in vitro* assays)	This paper	Table S4
**Recombinant DNA**		
pSL0007-pSL11244	This paper	Table S1
pKYA01-pKYA11	This paper	Table S1
**Software and algorithms**		
EPU software	Thermo Fisher Scientific	https://www.thermofisher.com/jp/en/home/electron-microscopy/products/software-em-3d-vis/epu-software.html
cryoSPARC v5.0.2	Punjani et al.[60]	https://cryosparc.com/; RRID:SCR_016501
COOT	Emsley et al.[65]	https://www2.mrc-lmb.cam.ac.uk/personal/pemsley/coot/; RRID:SCR_014222
Boltz-2	Passaro et al.[66]	https://github.com/jwohlwend/boltz
Servalcat	Yamashita et al.[67]	https://github.com/keitaroyam/servalcat
ProSMART	Nicholls et al.[68]	https://www.ccp4.ac.uk/html/prosmart.html
MolProbity	Williams et al.[69]	https://www.phenix-online.org/documentation/reference/molprobity_tool.html; RRID:SCR_014226
Topaz	Bepler et al.[61]	https://github.com/tbepler/topaz
UCSF ChimeraX	Pettersen et al.[70]	https://www.rbvi.ucsf.edu/chimerax/; RRID:SCR_015872
CueMol	N/A	http://www.cuemol.org
Prism	Graphpad	https://www.graphpad.com/scientific-software/prism/

REAGENT or RESOURCE	SOURCE	IDENTIFIER
**Software and algorithms**		
Cutadapt v5.0	Martin[50]	https://cutadapt.readthedocs.io; RRID:SCR_011841
bwa-mem2 v2.2.1	Vasimuddin et al.[51]	https://github.com/bwa-mem2/bwa-mem2; RRID:SCR_022192
Bowtie2 v2.2.1	Langmead and Salzberg[73]	http://bowtie-bio.sourceforge.net/bowtie2; RRID:SCR_016368
SAMtools v1.17	Danecek et al.[52]	http://www.htslib.org; RRID:SCR_002105
deepTools (bamCoverage) v3.5.6	Ramírez et al.[53]	https://deeptools.readthedocs.io; RRID:SCR_016366
featureCounts (Subread) v2.0.2	Liao et al.[55]	https://subread.sourceforge.net; RRID:SCR_012919
PyDESeq2 v0.5.3	Muzellec et al.[56]	https://github.com/owkin/PyDESeq2
gseapy v1.1.13	Fang et al.[77]	https://github.com/zqfang/GSEApy
bbduk (BBTools) v39.83	Bushnell[76]	https://sourceforge.net/projects/bbmap/
breseq v0.39.0	Deatherage and Barrick[75]	https://github.com/barricklab/breseq; RRID:SCR_010810
MEME v5.5.7	Bailey et al.[58]	https://meme-suite.org; RRID:SCR_001783
IGV v2.17.4	Robinson et al.[54]	https://igv.org; RRID:SCR_011793
MaxQuant v2.0.3.0	Cox and Mann[59]	https://www.maxquant.org; RRID:SCR_014485
Skyline-daily v24.1.1.398	MacLean et al.[79]	https://skyline.ms; RRID:SCR_014080
MMseqs2	Steinegger and Söding[41]	https://github.com/soedinglab/MMseqs2
MAFFT v7.490	Katoh and Standley[45]	https://mafft.cbrc.jp/alignment/software/; RRID:SCR_011811
FastTree 2 v2.1.11	Price et al.[43]	http://www.microbesonline.org/fasttree/; RRID:SCR_015501
iTOL	Letunic and Bork[44]	https://itol.embl.de; RRID:SCR_018174
mLocARNA v2.0.1	Will et al.[46]	http://www.bioinf.uni-freiburg.de/Software/LocARNA/
Infernal v1.1.4	Nawrocki and Eddy[47]	http://eddylab.org/infernal/; RRID:SCR_011809
CD-HIT	Fu et al.[48]	https://sites.google.com/view/cd-hit; RRID:SCR_007105
R-scape v2.6.10	Rivas et al.[49]	http://eddylab.org/R-scape/
DECIPHER v3.8.0	Wright[71]	https://bioconductor.org/packages/DECIPHER/; RRID:SCR_001357
WebLogo v3.7.9	Crooks et al.[72]	https://weblogo.threeplusone.com; RRID:SCR_010236
Xcalibur v4.5.474.0	Thermo Fisher Scientific	https://www.thermofisher.com/us/en/home/industrial/mass-spectrometry/liquid-chromatography-mass-spectrometry-lc-ms/lc-ms-software/lc-ms-data-acquisition-software/xcalibur-data-acquisition-interpretation-software.html; RRID:SCR_014593
**Other**		
Ni-NTA Superflow resin	QIAGEN	Cat#30450
HiTrap Heparin HP column	GE Healthcare	Cat#17040601
Superdex 200 Increase 10/300 column	GE Healthcare	Cat#28990944
Amicon Ultra-4 mL Centrifugal Filter Unit (MWCO 30 kDa)	Merck Millipore	Cat#UFC803008
Amicon Ultra-0.5 mL Centrifugal Filter (MWCO 3 kDa)	Merck Millipore	Cat#UFC5003
Cu 300 mesh R1.2/1.3 grid covered with a 2 nm carbon film	Quantifoil	https://www.quantifoil.com/products/quantifoil/quantifoil-circular-holes/
Protein G Dynabeads	Thermo Fisher Scientific	Cat#10004D
